# A Camphorsulfonic Acid-Grafted Polybenzimidazole Ion Selectivity Membrane for Vanadium Redox Flow Battery

**DOI:** 10.3390/membranes15120374

**Published:** 2025-12-05

**Authors:** Yujie Guo, Bo Pang, Fujun Cui, Tingxu Fang, Li Tian, Liu Yang, Zeyu Chen, Xuemei Wu

**Affiliations:** 1 State Key Laboratory of Fine Chemicals, Research and Development Center of Membrane Science and Technology, School of Chemical Engineering, Dalian University of Technology, Dalian 116024, China; 2 Panjin Institute of Industrial Technology, Dalian University of Technology, Panjin 124221, China; 3 School of Chemical Engineering, Ocean and Life Sciences, Dalian University of Technology, Panjin 124221, China

**Keywords:** vanadium redox flow battery, camphorsulfonic acid grafting, ion selectivity, microphase separation, free volume engineering

## Abstract

The design of the chemical structure of ion-conductive membranes is critical to enhance proton/vanadium ion selectivity and the performance of vanadium redox flow batteries (VRFBs). Herein, camphorsulfonic acid is proposed as a novel proton-conductive group and grafted on polybenzimidazole (PBICa). The pendant sulfonic acid group on the end of the grafted side chains is flexible to promote the aggregation of ionic clusters at even a relatively low ion-exchange capacity (IEC) of 2.14 mmol g^−1^. The formation of these high-quality clusters underscores the remarkable efficacy of this structural strategy in driving nanoscale phase separation, which is a prerequisite for creating efficient proton-conducting pathways. The bulky and non-coplanar architecture of the camphorsulfonic acid group helps to increase the proportion of free volume compared with the conventional sulfonated polybenzimidazole, which not only promotes water uptake to facilitate proton transport but also exerts a sieving effect to effectively block vanadium ion permeation. The well-formed ionic clusters, together with the expanded free volume architecture, endow the membrane with both high proton conductivity (30.5 mS cm^−1^) and low vanadium ion permeability (0.15 × 10^−7^ cm^2^ s^−1^), achieving excellent proton/vanadium ion selectivity of 9.85 × 10^9^ mS s cm^−3^, which is about 5.6-fold that of a Nafion 212 membrane. Operating at 200 mA cm^−2^, the PBICa-based VRFB achieves an energy efficiency of 78.4% and a discharge capacity decay rate of 0.32% per cycle, outperforming the Nafion 212-based battery (EE of 76.9%, capacity decay of 0.79% per cycle).

## 1. Introduction

The deteriorating global energy crisis and environmental challenges are driving demands for clean, renewable, and efficient energy solutions [1,2,3,4,5]. Vanadium redox flow batteries (VRFBs) have emerged as a leading technology for large-scale renewable energy storage due to their high safety, superior energy efficiency, and environmental friendliness [6,7,8]. Operating in an aqueous environment containing multi-ion species, the high efficiency of VRFBs strongly relies on the ion-conductive membrane, which should have high proton conductivity [9,10], low vanadium ion permeability [11,12], and excellent chemical stability [13].

The design of the chemical structure of ion-conductive membranes is critical for high ion-selective conduction. Chemical modifications, such as increasing the amount of sulfonic acid ion-exchange groups [14,15,16], grafting hydrophilic sulfonated side chains [17,18,19], or introducing hydrophilic–hydrophobic blocks into polymer main chains [20,21,22], enhance microphase separation and form interconnected proton-conductive channels through the ionic clusters in membranes. However, commonly applied commercial polymers, such as Nafion and polybenzimidazole (PBI), usually possess tightly packed polymer chains, which limit the degree of microphase separation [23,24]. Excessive membrane swelling may occur with the increasing ion-exchange capacity (IEC), leading to serious vanadium ion permeation [25,26].

The design of macromolecular or non-coplanar structural units, either in polymer backbones or on side chains [27], can relax the interchain packing and increase the free volume between polymer chains, which increases water uptake and proton conductivity without causing significant membrane swelling. A significant challenge in the molecular design of high-selectivity ion-conductive membranes lies in the intrinsic limitation of conventional bulky structural units. State-of-the-art candidates, such as triphenylphosphonium [28] and naphthalene [29] groups, while effective for creating free volume and imparting steric hindrance, typically lack intrinsic proton-conductive functional groups (e.g., sulfonic or phosphoric acids [30,31]). This deficiency creates a critical bottleneck. More critically, the very steric bulk that enables size-sieving properties can physically separate the existing proton-conductive sites within the polymer matrix [32,33]. This disruption impedes the formation of continuous, well-connected proton-conductive pathways, which are essential for high proton mobility. Consequently, the incorporation of such non-conductive bulky units often comes at the cost of compromised proton conductivity, thereby fundamentally limiting the overall performance of the membrane and exacerbating the classic trade-off between selectivity and conductivity [34]. Very recently, advanced strategies focusing on the fabrication of rational membrane structures with precisely controlled microporous architectures have emerged, demonstrating exceptional proton selectivity [35]. This highlights the ongoing importance of tailoring free volume and nanoscale channels for high-performance VRFBs, a principle that our design directly embodies.

In this work, camphorsulfonic acid, acting as a novel non-coplanar proton-conductive group, was grafted on polybenzimidazole (PBICa) to enhance flow battery performance. In contrast to the conventional sulfonated polybenzimidazole (SPBI), where the sulfonic acid group directly attaches to the PBI backbone, the pendant camphorsulfonic acid group on the end of the grafted side chain is flexible. This configuration grants the sulfonate group significant conformational freedom, enhancing its mobility. This mobility is critical, as it enables the sulfonate groups to readily self-assemble and facilitates the dynamic reorganization of the polymer matrix. As a result, even at a relatively low ion-exchange capacity (IEC) of 2.14 mmol g^−1^, the system can spontaneously form well-defined, densely packed ionic clusters. The formation of these high-quality clusters underscores the remarkable efficacy of this structural strategy in driving nanoscale phase separation, which is a prerequisite for creating efficient proton-conducting pathways. Furthermore, the bulky, non-planar architecture of the camphorsulfonic acid group significantly disrupts the packing of polymer chains, leading to an expanded and more interconnected free volume compared to conventional SPBI. This unique microstructure serves a dual purpose: it facilitates the formation of well-hydrated proton transport channels, thereby enhancing proton conductivity, while simultaneously acting as a precise molecular sieve. The latter effect efficiently blocks the crossover of vanadium ions due to their larger hydrated ionic radius, thereby significantly boosting the membrane’s ion selectivity. At a low IEC of 2.14 mmol g^−1^, the PBICa membrane develops comparable ion cluster morphology to that of an SPBI membrane with much higher IEC (3.17 mmol g^−1^) while achieving significantly lower vanadium ion permeability (0.15 × 10^−7^ cm^2^ s^−1^). As a result, the PBICa membrane achieves a high proton/vanadium ion selectivity of 9.85 × 10^9^ mS s cm^−3^, about 6.0- and 5.6-fold that of SPBI and Nafion 212 membranes, respectively. At 200 mA cm^−2^, the PBICa-based battery shows an energy efficiency (EE) of 78.4% and a substantially lower discharge capacity decay rate of 0.32% per cycle, outperforming the Nafion 212-based battery (EE of 76.9%, capacity decay of 0.79% per cycle).

## 2. Materials and Methods

### 2.1. Materials

Poly(4,4′-diphenyl ether-5,5′-bisbenzimidazole) (PBI, M_n_ = 14,000 Da) was supplied by Shanghai Shengjun Plastic Technology Co., Ltd. (Shanghai, China), 3-bromocamphor-8-sulfonic acid ammonium salt was procured from Aladdin Reagent Co., Ltd. (Shanghai, China), and the Nafion 212 membrane was obtained from DuPont (USA). Anhydrous potassium carbonate, ethyl acetate, dimethyl sulfoxide (DMSO), and anhydrous ethanol were sourced from Tianjin Fuyu Fine Chemical Co., Ltd. (Tianjin, China). All chemicals were used as received without further purification.

### 2.2. Synthesis of Camphorsulfonic Acid-Grafted PBI (PBICa)

**Scheme 1:**
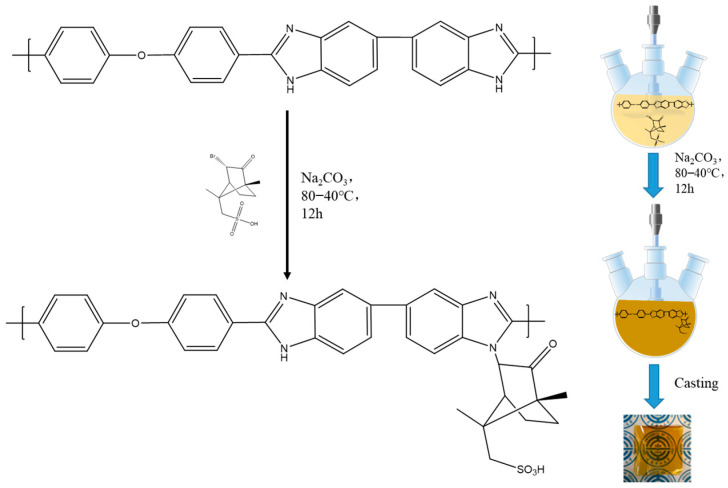
Synthesis of the camphorsulfonic acid-grafted polybenzimidazole (PBICa).

As shown in Scheme 1, 5 g of PBI was dissolved in 200 mL of anhydrous DMSO under an argon atmosphere. The solution was mixed with 13.5 g of anhydrous K_2_CO_3_ powder and stirred at 80 °C for 2 h. Subsequently, the solution was cooled to 40 °C and 6.5 g of ammonium 3-bromocamphor-8-sulfonate was added. The reaction proceeded for 18 h under stirring. The reaction product was precipitated by ethyl acetate, washed with deionized water and ethanol, and dried at 80 °C under vacuum to produce PBICa.

SPBI was synthesized via a direct sulfonation reaction. Briefly, 20 g of PBI was dissolved in 400 mL of concentrated sulfuric acid and the solution was kept at 150 °C for 6 h. After the reaction, the product was precipitated by deionized water, neutralized with concentrated sodium bicarbonate solution, thoroughly washed with deionized water until neutral, and dried overnight at 80 °C. The ion-exchange capacity (IEC) of SPBI, determined by acid–base titration, was 3.17 mmol g^−1^.

For fabrication of ion-conductive membranes by solution casting, the casting solution (1 wt% polymer in DMSO) was cast onto a clean glass plate and dried in an oven at 80 °C. The resulting membranes, with a controlled thickness of 20 μm ± 1.5 μm (measured by a digital micrometer at multiple points), were carefully peeled off for further testing.

### 2.3. Characterization

The chemical structure of the synthesized polymers was characterized by nuclear magnetic resonance (^1^H NMR, Varian Unity Inova (Agilent Technologies, Santa Clara, CA, USA) spectrometer with dimethyl sulfoxide-d_6_ as the solvent) and Fourier transform infrared (FTIR, Bruker Equinox 55 (Bruker, Billerica, MA, USA) spectrometer in the wavenumber range of 600 to 4000 cm^−1^) spectroscopy. The degree of sulfonation (DS) was determined by ^1^H-NMR and the IEC calculated using Equation (1), where ${\bar{M}}_{PBICa}$ and ${\bar{M}}_{PBI}$ are the molar mass of repeating units in PBICa and the pristine PBI, respectively.

(1) $$IEC=\frac{DS}{DS\times {\bar{M}}_{PBICa}+\left(1-DS\right)\times {\bar{M}}_{PBI}}$$

The membrane morphology was characterized by scanning electron microscopy (SEM; JSM 6300F (Jeol, Tokyo, Japan)) and transmission electron microscopy (TEM; JEM-2000EX (Jeol, Tokyo, Japan)). The TEM samples were immersed in a 0.5 M AgNO_3_ solution at room temperature for at least 48 h prior to imaging. The mechanical property of the membranes was evaluated using a universal testing machine (SANS CMT8102 (Shenzen, China)) at a constant crosshead speed of 5 mm min^−1^.

The vanadium ion permeability of the membrane was evaluated using a diffusion cell filled with a certain amount (*V*) of 1.5 M VOSO_4_ solution in 3 M H_2_SO_4_ and 1.5 M MgSO_4_ in 3 M H_2_SO_4_, respectively in the donor and receptor compartment. The concentration of VO^2+^ ions in the receptor compartment with time (*C_B_*) was monitored at 12 h intervals using a UV-vis spectrophotometer (PERSEE, Beijing, China) by measuring the absorbance at 760 nm, and calculated through a preestablished calibration curve. The vanadium ion permeability (P) was then calculated using Equation (2):

(2) $$V_{B}\frac{d\left(C_{B}\left(t\right)\right)}{dt}=A\frac{P}{L}\left(C_{A}-C_{B}\left(t\right)\right)$$

The area resistance (*AR*) of the membrane was measured using an electrochemical workstation (Ivium Technologies, Eindhoven, The Netherlands). Prior to testing, the membrane was equilibrated by immersion in a 1 M H_2_SO_4_ solution for 24 h. The measurement was carried out in an H-type diffusion cell at room temperature, with both compartments filled with 3 M H_2_SO_4_ aqueous solution. Electrochemical impedance spectroscopy (EIS) was performed over a frequency of 1–10^6^ Hz, both with and without the membrane assembled in the cell. The area resistance was then calculated using Equation (3):

(3) $$AR=\left(R_{1}-R_{2}\right)S$$

The ex situ chemical stability of the PBICa membrane was evaluated by immersing a sample (4 cm × 4 cm) in 60 mL of an oxidizing solution containing 1.5 M VO_2_^+^ in 3 M H_2_SO_4_ at room temperature for 40 days. The visual appearance of the membrane was documented before and after the immersion test. The concentration of VO_2_^+^ in the solution was monitored every 5 days using UV-vis spectrophotometry by measuring the absorbance at 760 nm.

The battery performance was evaluated using a Blue River battery test system (CT2001A, 5 V/2 A, LANDT, Vestal, NY, USA). A single cell was assembled with the 1 M H_2_SO_4_ solution-pretreated membrane (effective area: 9 cm^2^) and tested at room temperature. The negative and positive electrolytes consisted of 40 mL of 1.5 M V^2+^/V^3+^ in 3 M H_2_SO_4_ and 40 mL of 1.5 M VO^2+^/VO_2_^+^ in 3 M H_2_SO_4_, respectively. The charge–discharge cycling was performed between voltage limits of 0.8 V and 1.65 V for 200 cycles at a constant current density of 200 mA cm^−2^. The coulombic efficiency (CE), voltage efficiency (VE), and energy efficiency (EE) were calculated according to the following equations:

(4) $$CE=\frac{\int I_{d}dt}{\int I_{c}dt}\times 100\%$$

(5) $$VE=\frac{\int V_{d}I_{d}dt}{\int V_{c}I_{c}dt}\times 100\%$$

(6) EE=CE×VE×100%

where *I_c_* and *I_d_* are the charge and discharge currents and *V_c_* and *V_d_* represent the charge and discharge voltages, respectively.

## 3. Results

### 3.1. Chemical Structure of PBICa

**Figure 1:**
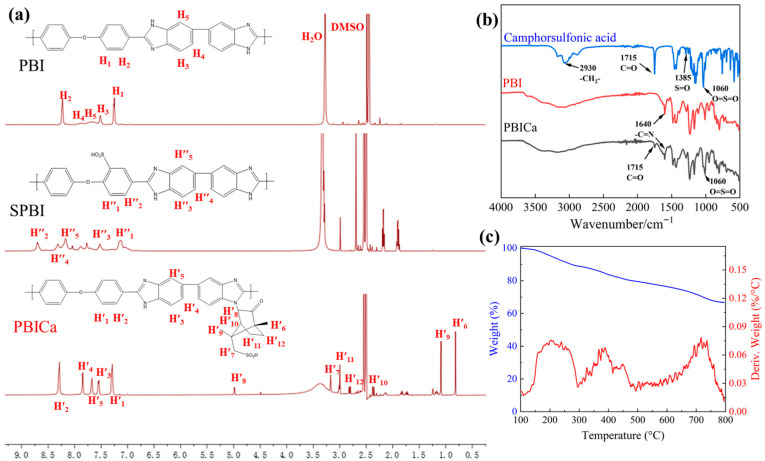
Chemical structure of the camphorsulfonic acid-grafted polybenzimidazole (PBICa). (**a**) ^1^H-NMR; (**b**) FTIR; (**c**) TGA.

A camphorsulfonic acid-grafted polybenzimidazole ion-conductive membrane, denoted PBICa, was designed and successfully synthesized. As shown in Figure 1a, the ^1^H NMR spectrum of PBICa shows several new peaks compared to that of the pristine PBI. In detail, the peaks appearing at chemical shifts of 0.8, 1.1, 2.3, 2.8, 3.0, 3.2, and 5.0 ppm are assigned to the protons of H_6_′, H_9_′, H_10_′, H_12_′, H_11_′, H_7_′, and H_8_′, respectively, in the camphorsulfonic acid side chain. The proton of H_8_ʹ, being close to the PBI backbone, is deshielded and appears downfield at 5.0 ppm. Furthermore, the substitution on the side chain shifts the signal of proton H_5_ on the aromatic ring from 7.7 ppm in PBI to 7.5 ppm in PBICa. The degree of substitution of the camphorsulfonic acid side chain (DS) was calculated from the area integral (S) of the peaks for aromatic protons H_1_ and H_1_’ using the equation DS = S(H_1_′)/[S(H_1_) + S(H_1_′)], giving a DS value of about 110%. Accordingly, the corresponding IEC was determined to be 2.14 mmol g^−1^ using Equation (1).

The successful synthesis of PBICa was further verified by FTIR spectroscopy. As presented in Figure 1b, the FTIR spectrum of the PBICa membrane not only retains the characteristic C=N stretching band (1640 cm^−1^) of the PBI backbone, but also reveals several new absorption bands attributable to the grafted camphorsulfonic acid side chains, including bands at 2930 cm^−1^ (symmetric –CH_2_- vibration), 1715 cm^−1^ (C=O stretching), 1385 cm^−1^ (S=O symmetric vibration), and 1060 cm^−1^ (O=S=O symmetric vibration). The appearance of these distinctive functional group vibrations is consistent with the chemical structure of camphorsulfonic acid. Therefore, the successful graft of the camphorsulfonic acid on the PBI backbone is confirmed by ^1^H NMR and FTIR characteristics.

### 3.2. Microstructure of PBICa

**Figure 2:**
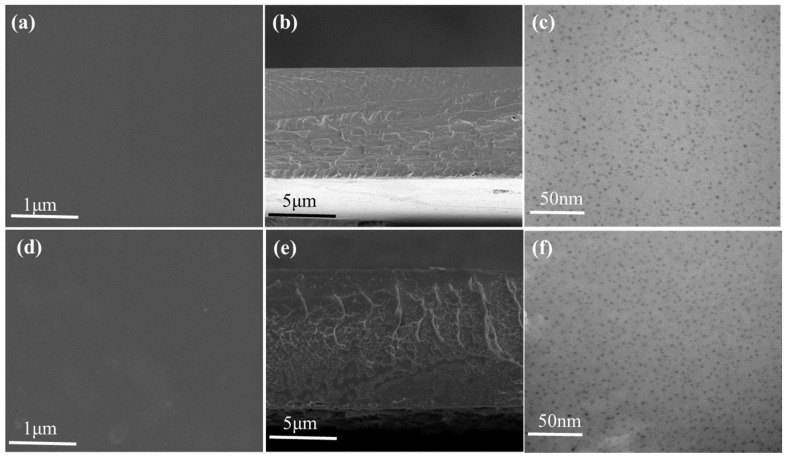
Comparative microstructural morphology of the sulfonated polybenzimidazole (SPBI, IEC of 3.17 mmol g^−1^) and camphorsulfonic acid-grafted polybenzimidazole (PBICa, IEC of 2.14 mmol g^−1^) membranes. (**a**–**c**) SPBI membrane: SEM images (**a**) surface, (**b**) cross section, and (**c**) TEM image. (**d**–**f**) PBICa membrane: SEM images (**d**) surface, (**e**) cross section, and (**f**) TEM image.

The SEM images shown in Figure 2a,b,d,e confirm that the PBICa membrane possesses a uniform and defect-free morphology in both surface and cross section compared to that of the sulfonated polybenzimidazole (SPBI, with the sulfonic acid group directly binding on the benzene ring of PBI). TEM images further reveal the well-defined microphase-separated structure of the PBICa membrane. As shown in Figure 2c,f, the hydrophilic ionic clusters (dark regions) are uniformly dispersed within the hydrophobic polymer matrix (bright regions). Notably, despite being at a lower IEC (2.14 mmol g^−1^), the PBICa membrane exhibits comparable ion cluster dimensions (~8–10 nm) to those of the high-IEC SPBI membrane (3.17 mmol g^−1^). This indicates a superior capability of the pendent camphorsulfonic acid groups for microphase separation. These interconnected ionic clusters will serve as the primary pathways for proton conduction in typically dense membranes. Additionally, the formation of a well-developed ionic cluster network at a lower IEC level can restrict excessive membrane swelling, which will effectively hinder the permeation of vanadium ions across the membrane.

**Figure 3:**
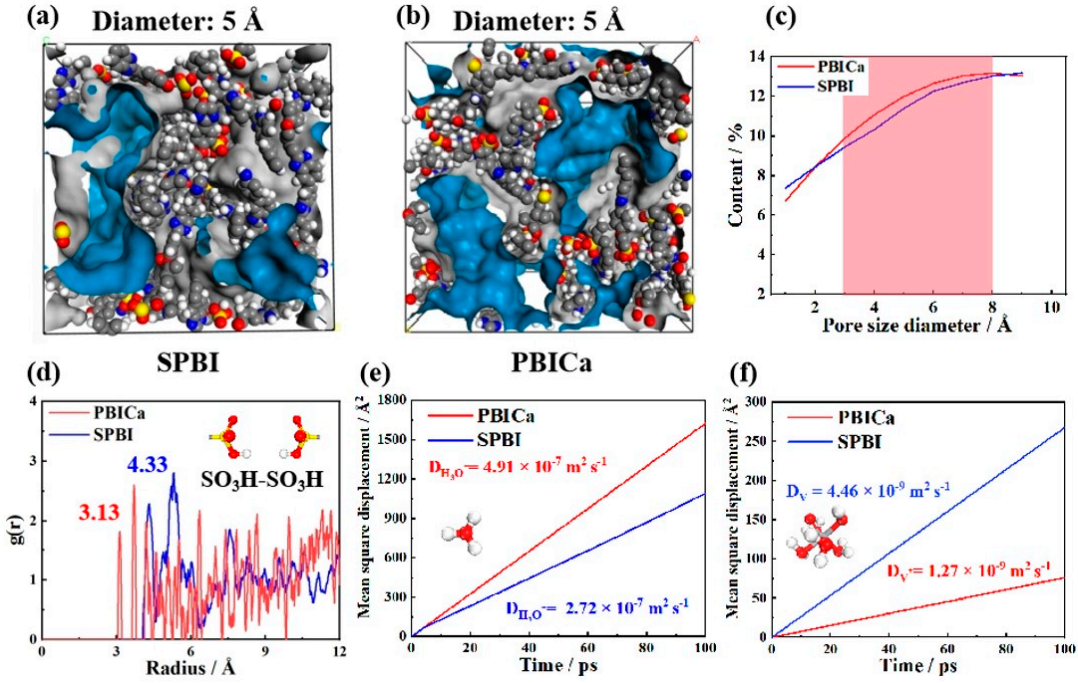
Molecular dynamic simulation. Free volume of (**a**) SPBI and (**b**) PBICa; (**c**) fractional free volume distribution; (**d**) radial distribution function of sulfonate groups; (**e**) calculated proton diffusion coefficient; (**f**) calculated vanadium ion diffusion coefficient.

Molecular dynamic simulation provides further insight into the microstructure of PBICa. The graft of camphorsulfonic acid on PBI demonstrates not only stronger ability to promote microphase separation, but also larger free volume fraction compared to the conventional SPBI (Figure 3a,b). Analysis of the free volume distribution reveals that PBICa possesses significantly higher proportion of pores with diameters between 3 and 8 Å (Figure 3c), which allows protons (with hydrated diameter less than 3 Å) through, but block vanadium ions (with hydrated diameter larger than 8 Å). This expanded free volume architecture is derived from the bulky, non-planar structure of the camphorsulfonic acid group, which disrupts the dense packing of polymer chains and introduces an additional pathway for selective proton conduction.

Radial distribution function (RDF) analysis indicates a closer proximity between the sulfonic acid groups in PBICa, with a characteristic peak at 3.13 Å versus 4.33 Å in SPBI (Figure 3d). This indicates the enhanced capability of microphase separation owing to the flexibility of camphorsulfonic acid side chain, which coincides with the comparable size of ionic clusters formed in PBICa, even at lower IEC. The well-formed ionic clusters together with the expanded free volume will help to promote water uptake and proton conduction. Consequently, the PBICa membrane achieves a proton diffusion coefficient of 4.91 × 10^−7^ m^2^ s^−1^, which is 1.8-fold that of SPBI (Figure 3e). A notable decrease in the vanadium ion diffusion coefficient is shown in Figure 3f, from 4.46 × 10^−9^ m^2^ s^−1^ in SPBI to 1.27 × 10^−9^ m^2^ s^−1^ in PBICa. These results provide conclusive evidence that the meticulously engineered free volume architecture within the PBICa membrane plays a decisive role in its performance. This architecture gives rise to a pronounced molecular sieving effect, which is highly effective in blocking the permeation of larger vanadium ions, as quantitatively demonstrated by the exceptionally low vanadium permeability of 0.15 × 10^−7^ cm^2^ s^−1^. The efficacy of this sieving mechanism is further underscored by the significant 91.4% reduction in permeability compared to the SPBI benchmark. Crucially, this selective barrier does not compromise proton transport. Instead, the interconnected free volume network facilitates the rapid conduction of protons, leading to a high diffusion coefficient of 4.91 × 10^−7^ m^2^ s^−1^. It is this synergistic combination—high proton mobility coupled with strongly suppressed vanadium ion crossover—that is the fundamental origin of the PBICa membrane’s outstanding ion selectivity, measured at an ultrahigh value of 9.85 × 10^9^ mS s cm^−3^. This synergistic mechanism successfully decouples the traditionally conflicting properties of conductivity and selectivity, establishing a new design paradigm for high-performance ion-conductive membranes.

### 3.3. Fundamental Properties of the PBICa Membrane

**Figure 4:**
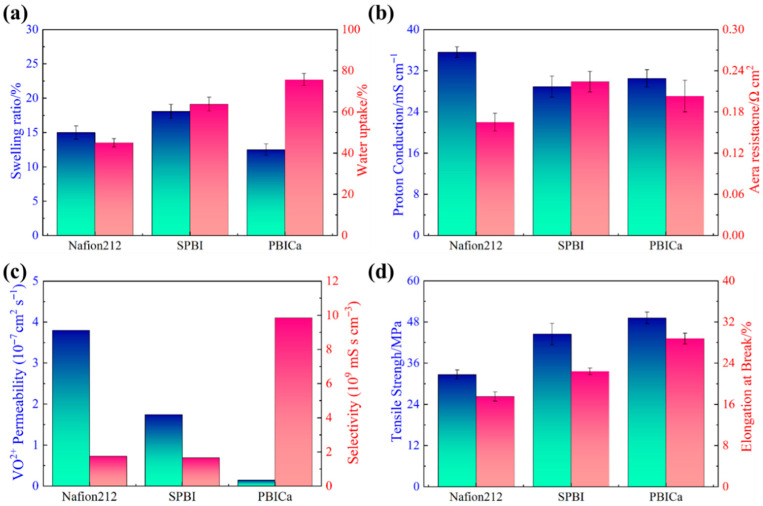
Fundamental properties of the PBICa, SPBI, and Nafion 212 membrane. (**a**) Water uptake and swelling ratio; (**b**) proton conductivity and area resistance; (**c**) vanadium ion permeability and ion selectivity; (**d**) tensile strength and elongation at break.

The fundamental properties of the PBICa, SPBI and Nafion 212 are listed in Table 1. The bulky, non-planar camphorsulfonic acid groups in PBICa generate substantial free volume, which enables the membrane to achieve higher water uptake (75.7%), even at a lower IEC compared to that of SPBI, thereby facilitating proton conduction (Figure 4a). Concurrently, the lower water swelling ratio (12.5%) of PBICa contributes to its enhanced vanadium ion-blocking capability and mechanical robustness. Despite the lower IEC, the PBICa membrane exhibits a slightly higher proton conductivity (30.5 mS cm^−1^) and a lower area resistance (0.203 Ω cm^2^) compared to SPBI with comparable ionic cluster size (28.9 mS cm^−1^ and 0.224 Ω cm^2^, respectively, Figure 4b). More significantly, the steric hindrance and proton/vanadium ion-screenable free volume imposed by the camphorsulfonic acid groups drastically reduce vanadium ion permeation. The vanadium ion permeability of the PBICa membrane is 0.15 × 10^−7^ cm^2^ s^−1^, which is 91.4% lower than that of the SPBI membrane (1.74 × 10^−7^ cm^2^ s^−1^) (Figure 4c). The combination of the efficient proton conduction and exceptional vanadium ion-blocking capability endows the PBICa membrane with a remarkable ion selectivity of 9.85 × 10^9^ mS s cm^−3^, which is 6.0- and 5.6-fold that of the SPBI membrane (1.65 × 10^9^ mS s cm^−3^) and Nafion 212 (1.76 × 10^9^ mS s cm^−3^), respectively. Furthermore, the rigid polymer backbone combined with the buffer effect of the free volume provide the PBICa membrane with higher strength and toughness compared to SPBI, as evidenced by a tensile strength of 49.2 MPa and an elongation at break of 28.8% (Figure 4d). The membrane also demonstrated excellent ex situ oxidative stability, as will be discussed in Section 3.4, which is crucial for long-term VRFB operation. The thermal stability of the membranes was evaluated by TGA (Figure 1). All membranes exhibited excellent thermal stability, with the major polymer backbone degradation occurring above 200 °C, confirming their suitability for VRFB applications.

### 3.4. Vanadium Redox Flow Battery Performance

**Figure 5:**
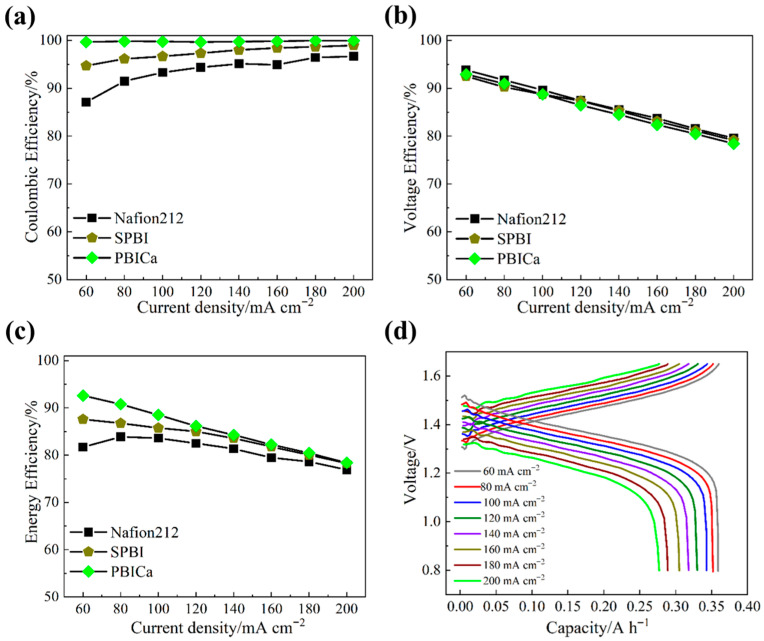
Performance of vanadium redox flow batteries assembled with the PBICa membrane, evaluated at different current densities. (**a**) Coulombic efficiency (CE); (**b**) voltage efficiency (VE), (**c**) energy efficiency (EE); (**d**) representative charge–discharge curves.

High proton/vanadium ion selectivity endows the PBICa membrane with excellent vanadium redox flow battery performance. As shown in Figure 5a, the battery assembled with the PBICa membrane maintains a high coulombic efficiency (CE) exceeding 99.5% across a wide range of current densities (60–200 mA cm^−2^). The consistently high CE (99.9%) for the PBICa membrane is a direct result of its exceptionally low vanadium ion permeability. Notably, despite the lower IEC in PBICa, the PBICa-based battery achieves a voltage efficiency (VE) comparable to that of the SPBI-based battery (Figure 5b), owing to the better microphase separation and free volume microstructure. By integrating the CE and VE, the overall energy efficiency (EE) was determined. As shown in Figure 5c, the PBICa membrane enabled the highest EE, ranging from 92.6% at 60 mA cm^−2^ to 78.4% at 200 mA cm^−2^. This performance surpasses that of the Nafion 212 membrane (81.7–76.9%) and the SPBI membrane (87.6–78.2%), suggesting the superiority of the PBICa membrane.

**Figure 6:**
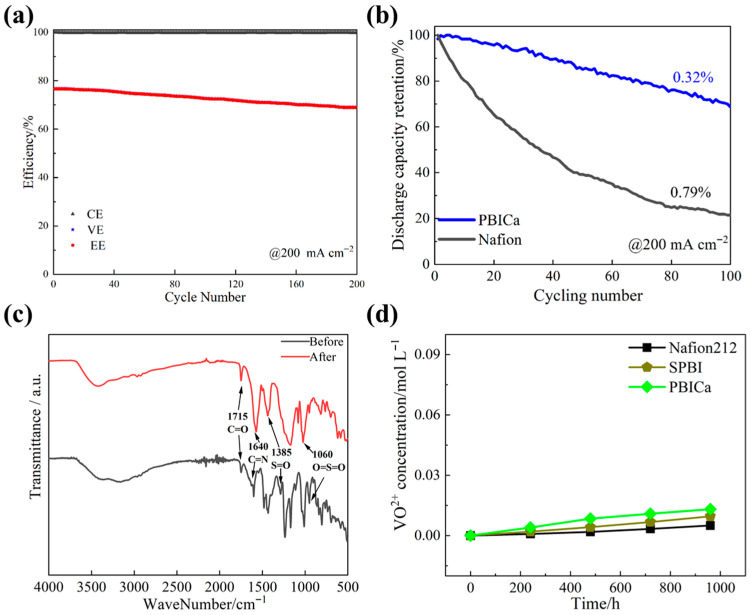
Cycling stability and chemical durability of the flow batteries. (**a**) Cycling stability over 200 cycles at 200 mA cm^−2^; (**b**) the corresponding capacity decay rate per cycle; (**c**) FTIR spectra of the membrane before and after cycling; (**d**) ex situ oxidative stability assessed by the evolution of VO^2+^ concentration during immersion in the VO^2+^ oxidizing solution.

During the cycling test at a high current density of 200 mA cm^−2^, the PBICa-based battery demonstrates good cycling stability (Figure 6a,b). It retained 68.7% of its initial discharge capacity after 100 cycles, corresponding to a low discharge capacity decay rate of 0.32% per cycle. In contrast, the battery assembled with the Nafion 212 membrane exhibits rapid capacity decay, retaining only 21.5% of its initial capacity with a discharge decay rate of 0.79% per cycle. Post-cycling FTIR analysis further confirms the good chemical stability of the PBICa membrane. The peaks of characteristic C=O stretching (1715 cm^−1^), C=N stretching (1640 cm^−1^), S=O stretching (1385 cm^−1^), and O=S=O stretching (1060 cm^−1^) show no noticeable shift, indicating an intact chemical structure of the membrane after cycling (Figure 6c). Moreover, the PBICa membrane shows high oxidative stability, as evidenced by the ex situ stability test result (Figure 6d). After 960 h immersion in the VO_2_^+^ oxidizing solution, the change in VO^2+^ concentration with time for PBICa was comparable to that for the SPBI and Nafion 212 membranes, confirming its excellent resistance to oxidative degradation.

## 4. Conclusions

This paper reports a novel polybenzimidazole-based ion-conductive membrane (PBICa) in which the bulky and non-planar camphorsulfonic acid was grafted as side chains on PBI backbone. The camphorsulfonic acid moiety can increase the free volume of PBICa owing to its bulky and non-planar configuration, which not only promotes water uptake to facilitate proton transport, but also exerts a sieving effect to effectively block vanadium ion permeation. Notably, the PBICa membrane, with a lower IEC of 2.14 mmol g^−1^, forms ionic clusters of a size (~8–10 nm) comparable to the SPBI membrane with a much higher IEC of 3.17 mmol g^−1^. The formation of these high-quality clusters underscores the remarkable efficacy of this structural strategy in driving nanoscale phase separation, which is a prerequisite for creating efficient proton-conducting pathways. The abundant free volume in PBICa facilitates higher water uptake (75.7%) and proton conductivity (30.5 mS cm^−1^) with a much lower swelling ratio (12.5%) and vanadium ion permeability (0.15 × 10^−7^ cm^2^ s^−1^), achieving ultrahigh ion selectivity of 9.85 × 10^9^ mS s cm^−3^. Consequently, the PBICa membrane exhibits excellent VRFB performance, with energy efficiency of 78.4% and a discharging capacity decay rate of 0.32% per cycle at 200 mA cm^−2^. This overall performance surpasses that of the commercial Nafion 212 benchmark (EE: 76.9%; decay rate: 0.79% per cycle). This work offers valuable guidance to chemical structural design for ion conduction promoted by both microphase separation and free volume, which is effective for high-performance energy storage applications.

## Figures and Tables

**Table 1:** Comparison of the PBICa, SPBI, and Nafion 212 membranes.

Membranes	PBICa	SPBI	Nafion 212
Thickness (μm)	20.6	21.3	50.8
Water uptake/swell ratio (%)	75.7/12.5	63.8/18.1	45.0/15.2
Area resistance (Ω cm^−2^)	0.20	0.22	0.17
Proton conduction/mS cm^−1^	30.5	28.9	35.6
Tensile strength/MPa	49.3	44.5	32.7
Vanadium permeability (10–7 cm^2^ s^−1^)	0.15	1.7	3.8
H+/Vn+ selectivity (109 mS s cm^−3^) (H+/Vn+ selectivity = (proton conduction/vanadium permeability)	9.9	1.7	1.8
CE/EE (200 mA cm^−2^)	99.9/78.4	98.9/78.3	96.7/76.9
Discharge capacity decay rate (200 mA cm^−2^%/cycle)	0.32	\	0.79

## Data Availability

The original contributions presented in this study are included in the article. Further inquiries can be directed to the corresponding author(s).
